# Accuracy of Diagnostic Investigations in Monitoring Hepatitis B Virus Infection: Strengths, Limitations, and Emerging Biomarkers

**DOI:** 10.3390/ijms27052464

**Published:** 2026-03-07

**Authors:** Laura Iulia Bozomitu, Ancuta Lupu, Vasile Valeriu Lupu, Nicoleta Gimiga, Dana Teodora Anton Paduraru, Dana Elena Mîndru, Mihaela Mihai, Carmen Anton, Emil Anton, Mihaela Mitrea, Anca Adam-Raileanu, Lorenza Forna

**Affiliations:** 1 Grigore T. Popa University of Medicine and Pharmacy Iasi, 700115 Iasi, Romania; laura.bozomitu@gmail.com (L.I.B.); valeriulupu@yahoo.com (V.V.L.); dana.anton@umfiasi.ro (D.T.A.P.); mindru.dana@umfiasi.ro (D.E.M.); mihaela.grigoriu@umfiasi.ro (M.M.); carmen.anton@umfiasi.ro (C.A.); emil.anton@yahoo.com (E.A.); mihaela.mitrea77@gmail.com (M.M.); anca.adam60@gmail.com (A.A.-R.); lorenza.donea@yahoo.ro (L.F.); 2 Pediatrics—“Sf. Maria” Clinical Emergency Children Hospital, 700309 Iasi, Romania

**Keywords:** hepatitis B virus, biomarkers, immunological response, accuracy, infection

## Abstract

In October 2020, the International Coalition to Eliminate Hepatitis B Virus (ICE-HBV) updated the biomarker framework; they underscored major advances in the understanding of viral and immunologic markers, yet highlighted persistent gaps in their clinical integration. This is particularly the case in low- and middle-income regions, where HBV remains a substantial public health problem, including in the pediatric population. To synthesize contemporary evidence, a structured literature search was performed across PubMed/MEDLINE, Scopus, and Web of Science. Classical biomarkers—including HBeAg, HBV DNA, and quantitative HBsAg—remain central for disease staging and therapeutic monitoring, while emerging markers enhance precision in risk stratification: HBcrAg, which correlates strongly with intrahepatic cccDNA activity and virological rebound after NA discontinuation; serum HBV RNA, which offers additional insight into transcriptional activity, which is particularly relevant for RNA-targeted therapies; and quantitative anti-HBc (qAnti-HBc), which reflects stronger humoral imprinting and more competent HBV-specific immune memory, and is consistently associated with fewer ALT flares and reduced virological rebound at end of treatment. Despite these advances, assay standardization, genotype-related variability, and limited pediatric data constrain broad clinical application. Integrating classical and emerging biomarkers into personalized therapeutic algorithms offers substantial potential for refining treatment decisions, predicting post-treatment outcomes, and advancing HBV elimination strategies in diverse clinical settings.

## 1. Introduction

Chronic hepatitis B virus (HBV) infection remains a significant global health burden, with an estimation of 250 million people worldwide chronically infected and over 800,000 deaths annually due to HBV-related complications such as cirrhosis and hepatocellular carcinoma. Despite the effective vaccination programs, certain regions in Europe and globally continue to report high HBV prevalence rates, including persistently infected cases in pediatric population—a reflection of both vertical transmission and of incomplete or ineffective postnatal immunoprophylaxis [1,2].

Monitoring HBV infection has evolved substantially over the last decade, with serological, virological, and immunological biomarkers playing a pivotal role in diagnosis, prognosis, and therapeutic follow-up. In October 2020, the International Coalition to Eliminate Hepatitis B Virus (ICE-HBV) introduced an updated classification and evaluation of HBV biomarkers, aiming to harmonize their clinical use and identify gaps in current understanding. This comprehensive update focused on the accuracy and predictive value of both classical biomarkers—such as HBsAg, HBeAg, anti-HBc, and HBV DNA—and emerging biomarkers, including quantitative HBsAg (qHBsAg), HBV RNA, HBcrAg, and immunological markers of T-cell and B-cell response [1,3].

However, in clinical practice, interpretation of these biomarkers remains challenging due to inter-assay variability, biological heterogeneity, and dynamic nature of viral-host interactions. Moreover, the translation of research findings into clinical algorithms is inconsistent across healthcare systems, particularly in countries with limited access to advanced molecular diagnostics.

## 2. Materials and Methods

To provide an up-to-date synthesis of the evidence, in this narrative review, a comprehensive literature search was undertaken across major biomedical databases to identify studies examining the clinical relevance of HBV biomarkers. More specifically, searches were performed in PubMed/MEDLINE, Scopus, and Web of Science for publications from 1996 to 2025. The selected time frame reflects the period following the initial characterization of key HBV molecular and serological biomarkers and their subsequent clinical application. Search terms included combinations of the terms “HBV biomarkers,” “qHBsAg,” “HBcrAg,” “anti-HBc,” “HBV RNA,” and “cccDNA surrogate markers,” with inclusion restricted to original studies, clinical trials, systematic reviews, and clinical guidelines that assessed diagnostic accuracy, prognostic relevance, or therapeutic applicability. Based on this body of literature, the present review aims to provide an integrated framework for HBV biomarkers by summarizing their biological basis, clinical relevance, limitations, and comparative value across disease staging, treatment monitoring, and emerging management strategies.

## 3. HBV Components

Hepatitis B virus (HBV) is a small, enveloped DNA virus, whose infectious virion, known as the Dane particle, consists of a double-shelled spherical structure with a lipid envelope embedded with hepatitis B surface antigens (HBsAg). Inside the envelope lies the nucleocapsid, which contains the relaxed circular DNA (rcDNA) genome and the viral polymerase protein. After its attachment to the host cell membrane, the virus enters the hepatocytes and is transported to the nucleus, where rcDNA is converted into covalently closed circular DNA (cccDNA)—a stable episomal template that drives transcription of the pre-genomic RNA (pgRNA) and of other viral mRNAs. The maintenance and the transcriptional activity of cccDNA are strongly supported by the HBx protein [4,5].

The HBV genome contains four overlapping open reading frames (ORFs)—P, C, S, and X—which encode seven distinct proteins. This compact and overlapping genomic organization is a hallmark of viral efficiency [6]:

- P ORF encodes the HBV polymerase, thus mediating reverse transcription of the pgRNA into rcDNA [7].
- The C gene region encodes the core protein (HBcAg), which forms the nucleocapsid, and the precore protein, which is processed through the secretory pathway to generate HBeAg [8].
- S ORF encodes the surface antigens (S, M, L-HBs), essential for virion assembly and immunogenicity.
- X ORF encodes HBx, a regulatory protein that transactivates the cccDNA transcription and modulates the host’s restriction factors [9,10].

The S protein comprises 226 amino acids; the M protein includes the S domain plus an additional PreS2 domain (55 aa), while the L protein extends further with a PreS1 domain of 108–119 amino acids, depending on genotype [11].

These surface antigens play essential roles in virion’s assembly, secretion, immunogenicity, and in viral entry into hepatocytes [12]. Their structural complexity and immune reactivity make them a central focus of biomarker research in chronic HBV infection, given their contribution to persistence and immune escape mechanisms.

### 3.1. Clinical and Serological Biomarkers of HBV Infection

The serological profile of hepatitis B virus (HBV) infection remains the cornerstone for diagnosis, disease staging, and therapeutic monitoring. Classical biomarkers—HBsAg, HBeAg, anti-HBc, and HBV DNA—define the major clinical phases of chronic HBV infection and guide treatment decisions. HBsAg is the principal marker of active infection, reflecting both viral replication and antigen production, derived from intrahepatic covalently closed circular DNA (cccDNA) or integrated HBV DNA [13,14]. Its persistence beyond six months defines chronic infection, while its quantitative decline during therapy indicates improved viral control. HBeAg, a secreted protein associated with high viral replication and infectivity, serves as an indicator of immune-tolerant or immune-active disease; its seroconversion to anti-HBe antibodies marks the transition to the inactive carrier phase [15,16].

Anti-HBc antibodies (IgM and IgG) signal host exposure and immune recognition of the viral core. The presence of anti-HBc IgM denotes acute or recent infection, whereas anti-HBc IgG persists for life and helps distinguish chronic infection from resolved exposure. Quantitative assays for both HBsAg and HBV DNA have enhanced clinical precision, enabling refined phase classification and assessment of antiviral efficacy. However, despite these advances, conventional serological markers offer limited insight into intrahepatic viral transcriptional activity or the status of host immune control. They do not fully capture the persistence of transcriptionally active cccDNA or the complex immunologic interactions that determine disease progression and treatment outcomes [17,18].

This diagnostic gap has prompted the emergence of the next-generation biomarkers—such as HBV RNA and HBcrAg—which directly reflect the transcriptional and translational output of cccDNA and provide a more accurate assessment of residual viral activity and relapse risk. These novel tools complement the traditional serological framework, bridging the gap between molecular virology and clinical decision making [19].

### 3.2. Emerging Viral and Host Immune—Related Biomarkers

An ideal biomarker should accurately reflect the underlying biological activity of the disease and provide clinically meaningful information regarding diagnosis, prognosis, and therapeutic response. It must exhibit high specificity and sensitivity, remaining independent of viral genotype or host-related variability. Additionally, it should be predictive of clinical outcomes, reproducible across laboratories, and easily measurable through non-invasive and cost-effective methods. Importantly, an ideal biomarker must demonstrate correlation with intrahepatic viral activity, particularly with cccDNA transcriptional status, while being accessible and reliable in both high-resource and resource-limited settings.

- HBV RNA and its relationship to cccDNA

The presence of the serum HBV RNA, first described in 1996, represents one of the most important advances in understanding the replication biology of chronic B hepatitis. HBV RNA in serum consists mainly of pre-genomic RNA (pgRNA) that is incompletely reverse-transcribed within nucleocapsids derived from intrahepatic cccDNA [19]. Its quantification therefore provides a functional surrogate of cccDNA transcriptional activity, reflecting the dynamic state of viral replication and persistence even when HBV DNA is undetectable during nucleos(t)ide analogue (NA) therapy [20,21]. In chronic infection, HBV DNA integration into the host genome constitutes an additional source of HBsAg production, contributing to sustained antigenemia and T-cell dysfunction that drives immune exhaustion and hepatocellular injury [22,23]. The microvascular and immunologic microenvironment of the liver, altered by fibrosis and cirrhosis, further modulates intrahepatic cytotoxic T-cell activation and differentiation. Understanding these immunopathogenic interactions has paved the way for therapeutic approaches that aim to modulate intrahepatic inflammation and to restore antiviral immune competence [24,25].

- HBcrAg and host-immune response HBV biomarkers

Among emerging biomarkers, the hepatitis B core-related antigen (HBcrAg) has gained particular attention. HBcrAg comprises three structurally related proteins—HBcAg, HBeAg, and a truncated precore protein of 22 kDa (p22Cr)—which share an identical 149-amino-acid sequence. Using monoclonal antibody–based assays, these components can be measured collectively [26,27]. Multiple studies have demonstrated a strong correlation between HBcrAg, serum HBV DNA, and intrahepatic cccDNA levels. Detectable HBcrAg signifies ongoing transcription from cccDNA and is useful in differentiating HBeAg-positive from HBeAg-negative disease phases [28,29]. Clinically, HBcrAg serves as a multipurpose biomarker enabling:

▪ The assessment of disease phase and replication intensity;
▪ The prediction of HBeAg seroconversion;
▪ The monitoring of the response and durability of NA therapy;
▪ The identification of the necroinflammatory activity and the reactivation risk with immunosuppression;
▪ The estimation of hepatocellular carcinoma (HCC) risk [30,31].

In addition to viral biomarkers that directly reflect cccDNA transcriptional activity, increasing attention has been directed toward host immune-response markers that capture immune competence and immune-mediated viral control. Quantitative anti-HBc (qAnti-HBc) represents a surrogate of cumulative antigen exposure and humoral immune imprinting and has been associated with the capacity to maintain viral suppression after treatment cessation. Together with viral transcriptional markers such as HBV RNA and HBcrAg, qAnti-HBc contributes to an integrated assessment of transcriptional persistence and immune control and is increasingly considered within composite endpoints of functional cure [32].

### 3.3. Overview of Viral Replication and Biomarker Origin

1. Entry and cccDNA formation—HBV enters the hepatocytes and converts relaxed circular DNA (rcDNA) into cccDNA, a stable nuclear episome.
2. Transcription and translation—cccDNA acts as a template for multiple transcripts (mRNAs and pgRNA), directing the synthesis of viral structural and regulatory proteins.
3. Replication and assembly—pgRNA is reverse-transcribed into rcDNA and assembled with capsid and envelope proteins (HBsAg) to form mature virions.
4. The transcriptional activity of cccDNA gives rise to circulating viral products, including HBsAg and HBeAg, as well as emerging biomarkers such as HBV RNA and HBcrAg, which are discussed in detail in this article [24,33].

### 3.4. HBV RNA Transcripts and RNA-Interference-Based Therapeutics

Beyond their role in viral protein synthesis, HBV RNA transcripts represent key functional intermediates that have recently emerged as direct therapeutic targets. HBV generates several messenger RNAs—pre-genomic (C), Pre-S, S, and HBx mRNAs—each encoding specific viral proteins.

- pgRNA serves as a template for both reverse transcription into rcDNA and the translation of the polymerase and core proteins.
- Pre-S mRNA encodes the large envelope protein (L-HBs, containing Pre-S1/Pre-S2/S domains).
- S mRNA gives rise to medium and small surface proteins (M-HBs and S-HBs).
- HBx mRNA produces the regulatory HBx protein, a potent modulator of cccDNA transcription and host–cell signaling [20,34].

These transcriptional intermediates have become direct therapeutic targets for RNA-interference (RNAi) strategies. Small interfering RNAs (siRNAs) and antisense oligonucleotides targeting HBV mRNAs—especially the X ORF—induce degradation of viral transcripts through the RNA-induced silencing complex (RISC). This approach results in broad suppression of viral protein expression, including HBsAg, and shows promise in achieving functional cure when combined with immune-modulating therapies [35,36].

## 4. Predictive Studies on Relapse After Nucleos(t)ide Analogue (NA) Withdrawal

### 4.1. HBV RNA and HBcrAg as Predictors of Virological Relapse

Recent research highlights the predictive value of HBV RNA and HBcrAg in assessing the risk of viral relapse following discontinuation of nucleos(t)ide analogue (NA) therapy. These biomarkers are particularly relevant in HBeAg-negative patients who maintain undetectable HBV DNA levels under long-term antiviral suppression but still harbor transcriptionally active intrahepatic cccDNA [37,38]. Both serum HBV RNA and HBcrAg act as sensitive indicators of residual viral transcription. Detectable levels despite DNA suppression signify ongoing cccDNA activity and correlate with subsequent ALT flares and HBV DNA reactivation after therapy withdrawal. Their combined measurement during both natural disease evolution and antiviral treatment provides insight into the residual replicative potential of HBV and supports personalized management strategies [38,39].

A landmark study by Carey et al. [40] showed that HBV pre-genomic RNA (pgRNA) and HBcrAg levels strongly predicted virological and biochemical relapse in HBeAg-negative individuals after NA cessation, outperforming HBV DNA alone. Together, these markers enhance the clinician’s ability to identify patients at risk of post-treatment reactivation.

Clinically, serum HBV RNA has been shown to:

1. More accurately reflect the transcriptional activity of intrahepatic cccDNA;
2. Serve as a predictor for relapse risk after treatment discontinuation;
3. Function as a monitoring indicator of antiviral efficacy;
4. Provide prognostic insights into outcomes such as HBeAg seroconversion and hepatocellular carcinoma (HCC) risk.

However, not all studies have confirmed a robust predictive performance of HBcrAg across clinical endpoints. A recent systematic review and critical appraisal reported substantial variability in its diagnostic accuracy, with false-positive rates of approximately 9% and false-negative rates ranging from 12% to 35%. While HBcrAg showed good performance for predicting HBeAg seroconversion and HBeAg-negative chronic hepatitis, its utility was limited for predicting relapses after treatment discontinuation and for HBsAg loss. Moreover, correlations with HBV DNA, HBV RNA, and intrahepatic cccDNA decreased during antiviral therapy, underscoring the need for combined biomarker strategies rather than reliance on HBcrAg as a standalone predictor. These findings highlight the importance of cautious interpretation and context-specific application of HBcrAg in clinical decision making [41].

### 4.2. The Ideal Therapeutic Goal: Toward Functional and Sterilizing Cure

The goal of HBV therapy is to eradicate the virus, defined as complete elimination of both intrahepatic cccDNA and integrated HBV-DNA—termed sterilizing cure.

Experimental strategies using CRISPR/Cas9-based endonucleases have been designed to specifically cleave conserved HBV DNA sequences, offering a theoretical path toward viral eradication. However, major safety concerns remain, necessitating extensive evaluation before clinical application [42,43]. In the nearer future, therapeutic innovations aim to achieve functional cure, characterized by durable HBsAg serum clearance and sustained viral remission without ongoing treatment. Functional cure correlates with a markedly lower risk of reactivation, of progression to cirrhosis or HCC, particularly when achieved at small ages [44,45].

### 4.3. Novel Antiviral and Immunomodulatory Strategies

Multiple next-generation antiviral and immune-based therapies are aimed to enhance HBsAg’s clearance and to reconstitute the host’s immune control:

- RNA interference (RNAi) therapies using siRNAs (small interfering RNA) can significantly reduce expression of the key viral proteins (HBsAg, HBcAg, HBeAg) by silencing transcripts derived from the HBV X ORF, thereby mitigating immune exhaustion [46,47].
- Capsid assembly modulators/inhibitors (CAMs) disrupt pgRNA encapsidation, cccDNA recycling, and the capsid formation, halting replication at multiple stages.
- Entry and release inhibitors, which limit viral spread and reduce antigenemia [48].
- Therapeutic vaccines, Toll-like receptor (TLR) agonists, and RIG-I activators which are under investigation to boost innate and adaptive immune responses [49,50]. Together, these emerging modalities aim to achieve a more durable immune control and to complement biomarker-guided treatment strategies.

### 4.4. Clinical Relevance of Serum HBV RNA in Monitoring Viral Replication and Treatment Outcomes

In the clinical setting, quantification of the serum HBV RNA provides a functional readout of ongoing pre-genomic RNA synthesis, and residual cccDNA transcriptional activity [51]. Even when the HBV DNA becomes undetectable during long-term NA therapy, transcription from cccDNA may persist, and serum HBV RNA remains a reliable marker of residual replication [52,53].

Thus, HBV RNA represents an essential biomarker for:

- Monitoring active viral replication;
- Assessing antiviral efficacy;
- Guiding NA discontinuation;
- Evaluating a functional cure—patients with undetectable HBV RNA at the end of therapy exhibit a significantly lower risk of relapse, whereas persistent low-level positivity suggests ongoing transcriptional activity. Consequently, HBV RNA measurement bridges the diagnostic gap between biochemical remission and the true viral silence [54,55].

Beyond its role as a static end-of-treatment marker, longitudinal monitoring of serum HBV RNA offers additional clinical insight by distinguishing pharmacologic viral suppression from durable functional cure. Dynamic changes in HBV RNA levels may precede virological rebound or alanine aminotransferase (ALT) flares and can therefore inform post-therapy surveillance strategies. In clinical trial settings, HBV RNA is increasingly evaluated as a surrogate endpoint for therapeutic response, particularly in studies assessing RNA-targeted or immunomodulatory agents [56,57].

Despite its potential as a surrogate marker of intrahepatic cccDNA transcriptional activity, the clinical use of circulating HBV RNA is constrained by several biological and methodological limitations. First, the biological heterogeneity of circulating HBV RNA—including different genomic and sub-genomic species, as well as encapsidated and unencapsidated forms—complicates result interpretation. Second, the absence of an international quantification standard has led to the use of diverse in-house assays with different amplification targets and analytical sensitivities, resulting in inconsistent thresholds and variable predictive performance across studies. In addition, a proportion of circulating HBV RNA may originate from integrated HBV DNA, potentially overestimating cccDNA activity. Correlations between serum HBV RNA and intrahepatic cccDNA vary according to disease phase, patient characteristics, and antiviral treatment status. Finally, limited assay availability, lack of regulatory approval for routine clinical use, and restricted access outside research settings currently hinder widespread implementation. Consequently, assay standardization, longitudinal validation, and clearer definition of clinically relevant scenarios are required before HBV RNA can be reliably integrated into routine clinical practice [19,58].

### 4.5. Quantitative HBsAg and HBV DNA as Dynamic Risk Stratifiers

Quantitative HBsAg and HBV DNA levels remain indispensable as dynamic biomarkers for:

- Monitoring response to pegylated interferon therapy;
- Estimating the likelihood of functional clearance of HBsAg;
- Predicting reactivation risk in immunosuppressed patients;
- Differentiating inactive carriers from those with low-level viral activity [59].

### 4.6. Predictive Biomarkers for Nucleos(t)ide Analogue (NA) Therapy Discontinuation

The decision to discontinue long-term nucleos(t)ide analogue (NA) therapy in patients with chronic hepatitis B remains one of the most challenging aspects of clinical management. Recent evidence emphasizes that a subset of carefully selected patients—particularly those with HBeAg-negative infection and long-term viral suppression—may safely stop antiviral therapy under close biomarker-guided monitoring. A growing body of research highlights the combined predictive utility of quantitative HBsAg (qHBsAg) and serum HBV-RNA as complementary indicators of residual intrahepatic viral activity and relapse risk [43,59].

In a prospective multicenter cohort, the authors [54] investigated 184 HBeAg-negative patients treated with entecavir for ≥2 years. The authors found that patients with low qHBsAg (<100 IU/mL) and undetectable HBV RNA at the time of NA cessation had a markedly reduced incidence of virological relapse and ALT flares compared to those with positive HBV RNA. The combination of these markers provided greater predictive accuracy than either biomarker alone, suggesting that HBV-RNA may serve as a real-time surrogate of cccDNA transcriptional activity under pharmacologic suppression.

Similarly, Carey et al. [40] reported that HBV pre-genomic RNA (pgRNA) and HBcrAg levels measured at the end of therapy were significant predictors of virological and biochemical relapse in HBeAg-negative individuals with stable NA suppression. Both markers were found to outperform HBV DNA as predictors of post-treatment reactivation, underscoring their clinical value for individualized discontinuation decisions.

Further supporting data come from Liu et al. [60], who analyzed 206 patients discontinuing NA therapy and demonstrated that combining HBV RNA, qHBsAg, and HBcrAg markedly improved relapse prediction compared to any single marker. The authors proposed an integrated model that achieved >80% specificity for sustained off-therapy remission.

Meta-analyses and guideline updates have since consolidated these findings. Lim SG et al. [61] emphasized that a multi-marker approach—incorporating qHBsAg, HBV RNA, HBcrAg, and possibly quantitative anti-HBc—offers the best predictive performance for functional cure. EASL 2025 [62] recommendations align with this concept, endorsing biomarker-guided NA discontinuation only when:

1. qHBsAg < 1000 IU/mL (ideally <100 IU/mL);
2. HBV RNA and HBcrAg are undetectable;
3. ALT levels remain stable under long-term suppression [62].

This integrated biomarker strategy reduces the risk for relapse, minimizes unnecessary drug exposure, and supports personalized therapeutic endpoints. Collectively, these studies converge on a biologically grounded model, where HBV RNA serves as the most dynamic marker of transcriptional persistence, while HBcrAg and qHBsAg provide additional layers of viral and immunologic insight.

## 5. Integrative Framework and Clinical Implications

### 5.1. Combined Stratification Scheme: Viral, Inflammatory, Fibrotic, and Risk Parameters

According to the EASL 2025 recommendations [62], a combined approach integrating the viral load, the inflammatory activity, the stage of fibrosis, and the risk profile is now recommended for an accurate staging of the disease and for management decisions. This four-dimensional assessment reflects the dynamic interplay between virological and host-related factors (Table 1).

*Practical implications include:*

- Initiation of therapy when HBV DNA > 2000 IU/mL + elevated ALT + fibrosis ≥ F2–F3;
- Periodic monitoring every 3–12 months for untreated patients, based on individual risk profiles;
- Discontinuation of NA therapy only when HBsAg < 1000 IU/mL, ideally combined with HBcrAg and/or HBV RNA assessment and close post-cessation follow-up.

Recent data support the additive diagnostic power of combining viral and metabolic biomarkers in the evaluation of chronic hepatitis B. In a prospective cohort study by Liang et al. [63], a receiver operating characteristic (ROC) curve analysis demonstrated that the combination of HBV RNA, HBV DNA, and methionine (Met) achieved the highest diagnostic performance for distinguishing chronic HBV infection, with an area under the curve (AUC) of 0.840 (*p* < 0.01). This performance was superior to that of any single marker alone, underscoring the synergistic value of the multimarker integration in the refining risk stratification and improving diagnostic accuracy.

The study suggests that coupling molecular virological indices (reflecting cccDNA transcription and replication) with metabolic parameters that mirror hepatocellular function can provide a more comprehensive understanding of the disease activity and transition across the clinical phases—from chronic infection to cirrhosis or hepatocellular carcinoma [64].

### 5.2. Anti-HBc Levels as a Predictor of Relapse

- Prospective evidence (2025): In a multicenter prospective cohort of patients stopping NA therapy, end-of-treatment (EOT) anti-HBc ≥ 300 IU/mL identified a subgroup with low risk of virological relapse and severe biochemical flares; none of the patients above this threshold experienced severe relapse (PPV 100% for “no severe flare,” NPV 48%) [65].
- Earlier prospective data (2019): A large study found that higher EOT anti-HBc independently associated with lower clinical relapse over long-term follow-up after NA cessation, supporting a protective immune correlate captured by qAnti-HBc [66].
- Cohort heterogeneity (2022–2025): Not all data sets align perfectly. An HBeAg-negative cohort (mostly genotypes B/C) reported lower anti-HBc levels linked to lower relapse risk, highlighting possible genotype, disease-phase, and assay differences; a 2025 analysis again emphasized HBV-RNA’s added value but also variability across platforms. These mixed findings argue for context-specific cut-offs rather than a single universal threshold [67].
- Guideline position (2025): EASL 2025 acknowledges qAnti-HBc as a useful adjunct—particularly at NA stop—but recommends multimarker approaches (HBV DNA, quantitative HBsAg, HBcrAg, ±HBV RNA) until prospective validation and standardization of qAnti-HBc cut-offs are finalized [62].

### 5.3. Functional Pathway

Higher qAnti-HBc likely reflects stronger humoral imprinting and, by association, more competent HBV-specific immune memory, which may restrain rebound when pharmacologic suppression is lifted. This aligns with the observations that patients with high anti-HBc at EOT have fewer ALT flares and a less virological rebound after NA stopping [65].

**Main points:**

- Evidence suggests that qAnti-HBc ≥ 300 IU/mL at EOT may be a favorable signal when contemplating NA discontinuation—only in combination with low qHBsAg, undetectable HBV RNA, and low/negative HBcrAg, plus stable ALT [65,68].
- Borderline qAnti-HBc values have shown variable associations with outcomes across studies, with differences influenced by viral genotype, HBeAg status, fibrosis stage, and assay methodology, supporting the need for context- and laboratory-specific interpretation rather than universal cut-offs [67].
- Current evidence and EASL 2025 guidance indicate that qAnti-HBc is best interpreted as an adjunct biomarker within multimarker frameworks, rather than as a standalone determinant of clinical decision making [62].

## 6. Occult HBV Infection

Occult hepatitis B virus infection (OBI) is defined by the absence of HBsAg in serum and the persistence of intrahepatic cccDNA and/or circulating HBV DNA. According to the updated international consensus from the Taormina workshop (2018), OBI represents a distinct virological entity characterized by extremely low viral replication, fluctuating or intermittently detectable viremia, and preserved transcriptional templates within hepatocytes [69,70,71]. Because of these features, accurate diagnosis of OBI, generally relies on repeated sampling at different time points and on the use of sufficiently large serum or plasma volumes (≥1 mL) for DNA extraction to improve detection reliability. Although most PCR-based assays currently used in diagnostic laboratories are effective, their limited sensitivity may lead to false-negative results. Historically, nested PCR has represented one of the earliest high-sensitivity amplification strategies, employing two sequential rounds of PCR with internal primer sets to enhance analytical sensitivity and specificity. This approach has been widely used in early OBI research and has contributed significantly to the identification of low-level viral persistence in patients with serologically silent infection. However, nested PCR is technically demanding and requires strict laboratory precautions to minimize contamination risks, which may limit its routine clinical applicability. More recently, droplet digital PCR (ddPCR) has emerged as a highly sensitive and precise molecular platform capable of absolute quantification of HBV DNA through sample partitioning into thousands of nanoliter-scale droplets followed by end-point amplification and digital signal analysis. Compared with conventional real-time PCR, ddPCR offers improved reproducibility and enhanced detection of extremely low-level viremia, making it particularly valuable in research settings and in complex clinical scenarios such as OBI [72,73]. While ddPCR represents a modern advancement in viral load assessment, nested PCR remains an important historically validated research tool, and both methodologies contribute to a more comprehensive understanding of HBV persistence at very low viral levels [73].

Recent studies have demonstrated that ddPCR can detect approximately 25–40% of samples that were tested negative by conventional real-time PCR, thus highlighting its superior analytical sensitivity in identifying low levels of HBV DNA. This finding has been particularly emphasized in investigations of HBV reactivation among patients with chronic HCV infection treated with direct-acting antivirals, where ddPCR was able to reveal occult or residual viral replication undetected by qPCR. Accordingly, ddPCR technology enables the detection of HBV DNA levels below 10 IU/mL, which remain undetectable by conventional diagnostic assays [74,75,76].

Beyond analytical sensitivity, recent experimental data further refine the biological definition of OBI by distinguishing replication-competent HBV from integrated viral sequences. Novel mono- or double-over-gap PCR primer strategies have been proposed to selectively amplify HBV relaxed circular DNA (rcDNA) and/or cccDNA, but not integrated HBV DNA, providing a more specific laboratory-based approach for identifying true OBI in future clinical and research settings [71,77].

In parallel, emerging biomarker studies provide additional insight into intrahepatic viral persistence in low-replication states. In a recent study, HBcrAg levels were significantly higher in HBeAg-positive compared with HBeAg-negative patients and showed strong correlations with serum HBV DNA, intrahepatic total HBV DNA, pre-genomic RNA (pgRNA), cccDNA levels, and overall transcriptional activity. Notably, patients with undetectable HBcrAg (<3 log U/mL) exhibited a substantially lower intrahepatic cccDNA burden and reduced cccDNA transcriptional activity. Moreover, within a subset of HBeAg-negative individuals, elevated HBcrAg levels were associated not only with increased intrahepatic viral activity but also with more advanced fibrosis and higher necroinflammatory scores. Collectively, these findings support the concept that HBcrAg serves as a surrogate marker of residual intrahepatic replication competence and transcriptional activity, even in the context of low or fluctuating viremia, and highlight its potential complementary role in characterizing occult or low-level HBV persistence as well as in evaluating therapeutic strategies targeting the cccDNA reservoir [78].

Together, these updated definitions and diagnostic strategies highlight that OBI is not merely a serological phenomenon but a molecularly defined condition requiring highly sensitive and specific assays. The integration of consensus-based criteria with advanced detection methods is essential for improving diagnostic accuracy and for better understanding the clinical implications of occult HBV persistence, particularly in immunosuppressed patients and in the context of viral reactivation.

## 7. Analysis of HBV Biomarkers Interpretation

Table 2 illustrates the clinical interpretation of the main HBV viral markers, which may hold significant predictive value.

### 7.1. Clinical Implications of HBV Biomarkers Across Age Groups: Pediatric Versus Adult Populations

The interpretation and clinical relevance of HBV biomarkers vary considerably between pediatric and adult populations, primarily due to differences in the mechanism of viral acquisition, immune response maturation, and natural history of infection. In children, biomarkers primarily support disease phase classification and prevention strategies, whereas in adults they guide treatment optimization, prediction of relapse after therapy discontinuation, and long-term risk stratification.

#### 7.1.1. Pediatric Population

- In children, especially those infected perinatally or during early childhood, HBV infection frequently evolves toward chronicity, given the immaturity of the immune system and the inability to mount an effective cytotoxic response against infected hepatocytes. In this setting, biomarkers play a decisive role in early detection, classification of the disease phase, and in treatment monitoring: HBsAg and HBeAg remain essential for the identification of chronic infection and for defining the immune-tolerant versus immune-active phase. HBV DNA quantification provides information about replication intensity, guiding the timing of therapeutic intervention [52,79].
- In recent years, novel biomarkers such as HBV RNA and HBcrAg have emerged as potential tools for monitoring intrahepatic viral activity (cccDNA transcription) in pediatric patients, particularly useful when liver biopsy is not feasible. From a preventive standpoint, biomarker-based screening of pregnant women with HBV infection is fundamental to prevent mother-to-child transmission (MTCT). HBeAg positivity or high HBV DNA levels during pregnancy indicate the need for antiviral prophylaxis (e.g., tenofovir) in the third trimester and postnatal immunoprophylaxis in the newborn, thus substantially reducing the perinatal transmission risk [80,81]. Overall, in children, biomarkers predominantly inform monitoring and prevention rather than relapse prediction or functional cure assessment.

#### 7.1.2. Adult Population

**Figure 1:**
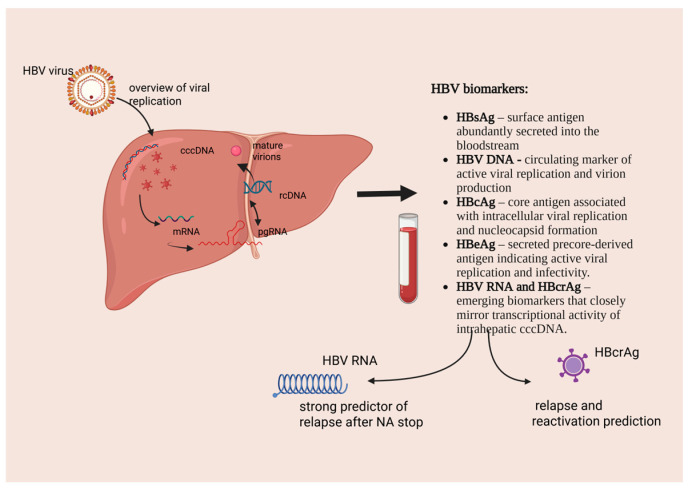
HBV biomarkers and overview of viral replication—Created in BioRender. Forna, L. (2025) https://app.biorender.com/illustrations/6914b1dc789dc6d0ae3c63f5 (accessed on 13 November 2025).

- In adults, HBV infection is more often acquired horizontally and has a higher likelihood of spontaneous resolution; however, once chronicity is established, it carries a greater risk of progressive liver injury. Unlike in children, biomarkers in this population have distinct implications for disease stratification, treatment optimization, and long-term surveillance: HBV DNA, HBeAg, and quantitative HBsAg remain standard tools for evaluating viral replication and treatment response with nucleos(t)ide analogue therapy [82,83]. Emerging biomarkers—particularly HBV RNA and HBcrAg—offer complementary insight into cccDNA transcriptional activity, providing early predictive value for sustained virological remission after treatment cessation (Figure 1).
- Quantitative decline of HBsAg and HBcrAg has been associated with functional cure and can assist in identifying patients eligible for treatment discontinuation. In individuals with advanced chronic liver disease, these markers contribute to the early detection and prevention of hepatocellular carcinoma (HCC), especially when integrated with imaging and serum AFP surveillance [84,85].

Thus, whereas pediatric biomarker interpretation emphasizes phase classification and prevention, adult biomarker interpretation focuses on therapeutic endpoints, relapse prediction, and long-term complication risk.

## 8. Conclusions

Accurate monitoring of hepatitis B infection requires a multidimensional approach integrating virological, immunological, and fibrotic markers. The ICE-HBV 2020 update and EASL 2025 guidelines jointly underscore the transition from static serology toward dynamic biomarker-based stratification.

Emerging indicators such as HBV-RNA and HBcrAg significantly improve the ability to predict the risk for relapse, the functional cure, and the treatment discontinuation safety, complementing the classic assays like HBsAg and HBV-DNA. The integration of the viral transcription markers with host’s inflammatory and fibrosis parameters provides a more complete representation of the disease activity and the therapeutic outcomes. While HBV-RNA quantification remains in a process of standardization, its “adoption”—together with quantitative HBsAg and HBcrAg—represents a pivotal step toward precision monitoring, functional cure, and ultimately, the elimination of HBV as a global public health threat.

From a clinical perspective, the relevance of both classic and emerging HBV biomarkers lies in their ability to provide a comprehensive and predictive overview of viral activity and host–virus interactions. Beyond their diagnostic and prognostic value, these biomarkers play a crucial role in preventing mother-to-child transmission, in the monitoring and optimization of antiviral therapy, and in the long-term maintenance of viral suppression to avoid disease flares or reactivation. Equally important, their predictive potential contributes to the early identification and prevention of hepatocellular carcinoma (HCC) in patients with chronic or advanced liver disease, thus guiding personalized surveillance and therapeutic strategies.

## Figures and Tables

**Table 1:** EASL 2025 Stratification Framework (adapted from EASL 2025 [62]).

Clinical Phase	Serological/Viral Features	Inflammation and Fibrosis	Interpretation
HBeAg-positive infection (non-inflammatory phase)	HBeAg^+^, HBV DNA markedly elevated, ALT normal	LSM < 7 kPa	Chronic infection without active hepatitis
HBeAg-positive hepatitis	HBeAg^+^, HBV DNA elevated, ALT increased	LSM ≥ 7–8 kPa	Active hepatitis with inflammation
Inactive HBeAg-negative carrier	HBeAg^−^, HBV DNA < 2000 IU/mL, ALT normal	LSM < 7 kPa	Inactive carrier phase
HBeAg-negative hepatitis	HBeAg^−^, HBV DNA > 2000 IU/mL, ALT elevated	LSM ≥ 7–8 kPa	Active hepatitis
HBsAg-negative phase (functional cure)	Loss of HBsAg, anti-HBs positive	-	Functional recovery

- Measns In the HBsAg-negative phase (functional cure), the infection is no longer active and fibrosis is no longer used to classify the phase.

**Table 2:** Interpretation of HBV biomarkers.

Marker	Reflects	Main Clinical Utility	Predictive Role
Anti-HBc	Host immune memory	Immune control marker	Lower relapse risk (≥300 IU/mL)
HBV DNA	Active replication	Diagnosis, treatment monitoring	Limited relapse prediction
HBsAg (quant.)	cccDNA + integrated DNA	Response to therapy, phase classification	Functional cure assessment
HBcrAg	Transcriptional cccDNA activity	Treatment response, HCC risk	Relapse and reactivation prediction
HBV RNA	cccDNA transcription	Detects residual replication	Strong predictor of relapse after NA stop

## Data Availability

No new data were created or analyzed in this study. Data sharing is not applicable to this article.

## References

[1] Chang M.H. (2008). Natural history and clinical management of chronic hepatitis B virus infection in children. Hepatol. Int..

[2] Forna L., Bozomitu L., Lupu A., Lupu V.V., Cojocariu C., Anton C., Girleanu I., Singeap A.M., Muzica C.M., Trifan A. (2024). Insights into the Natural and Treatment Courses of Hepatitis B in Children: A Retrospective Study. Biomedicines.

[3] Nguyen M.H., Wong G., Gane E., Kao J.H., Dusheiko G. (2020). Hepatitis B Virus: Advances in Prevention, Diagnosis, and Therapy. Clin. Microbiol. Rev..

[4] Locarnini S., Littlejohn M., Aziz M.N., Yuen L. (2013). Possible origins and evolution of the hepatitis B virus (HBV). Semin. Cancer Biol..

[5] Wu J.F., Tai C.S., Chang K.C., Chen Y.J., Hsu C.T., Chen H.L., Ni Y.H., Chang M.H. (2025). Predictors of Functional Cure of Chronic Hepatitis B Virus Infection: A Long-Term Follow-Up Study. Clin. Gastroenterol. Hepatol..

[6] Datta S., Chatterjee S., Veer V., Chakravarty R. (2012). Molecular biology of the hepatitis B virus for clinicians. J. Clin. Exp. Hepatol..

[7] Hu J., Seeger C. (1996). Expression and characterization of hepadnavirus reverse transcriptases. Methods Enzymol..

[8] McFadden W.M., Sarafianos S.G. (2023). Biology of the hepatitis B virus (HBV) core and capsid assembly modulators (CAMs) for chronic hepatitis B (CHB) cure. Glob. Health Med..

[9] Belloni L., Pollicino T., De Nicola F., Guerrieri F., Raffa G., Fanciulli M., Raimondo G., Levrero M. (2009). Nuclear HBx binds the HBV minichromosome and modifies the epigenetic regulation of cccDNA function. Proc. Natl. Acad. Sci. USA.

[10] Murphy C.M., Xu Y., Li F., Nio K., Reszka-Blanco N., Li X., Wu Y., Yu Y., Xiong Y., Su L. (2016). Hepatitis B Virus X Protein Promotes Degradation of SMC5/6 to Enhance HBV Replication. Cell Rep..

[11] Siegler V.D., Bruss V. (2013). Role of transmembrane domains of hepatitis B virus small surface proteins in subviral-particle biogenesis. J. Virol..

[12] Gish R.G., Given B.D., Lai C.L., Locarnini S.A., Lau J.Y., Lewis D.L., Schluep T. (2015). Chronic hepatitis B: Virology, natural history, current management and a glimpse at future opportunities. Antivir. Res..

[13] Liu J., Li T., Zhang L., Xu A. (2019). The Role of Hepatitis B Surface Antigen in Nucleos(t)ide Analogues Cessation Among Asian Patients With Chronic Hepatitis B: A Systematic Review. Hepatology.

[14] Kim S.W., Yoon J.S., Lee M., Cho Y. (2022). Toward a complete cure for chronic hepatitis B: Novel therapeutic targets for hepatitis B virus. Clin. Mol. Hepatol..

[15] Huang X., Hollinger F.B. (2014). Occult hepatitis B virus infection and hepatocellular carcinoma: A systematic review. J. Viral Hepat..

[16] Mak L.Y., Boettler T., Gill U.S. (2024). HBV Biomarkers and Their Role in Guiding Treatment Decisions. Semin. Liver Dis..

[17] Paganelli M., Stephenne X., Sokal E.M. (2012). Chronic hepatitis B in children and adolescents. J. Hepatol..

[18] Terrault N.A., Bzowej N.H., Chang K.M., Hwang J.P., Jonas M.M., Murad M.H., American Association for the Study of Liver Diseases (2016). AASLD guidelines for treatment of chronic hepatitis B. Hepatology.

[19] Deng R., Liu S., Shen S., Guo H., Sun J. (2022). Circulating HBV RNA: From biology to clinical applications. Hepatology.

[20] Choi W.-M., Choi J., Lim Y.-S., Seto W.-K., Eslam M. (2023). Chapter 7—Hepatitis B: Epidemiology, Natural History, and Diagnosis. Comprehensive Guide 403 to Hepatitis Advances.

[21] Martinez M.G., Villeret F., Testoni B., Zoulim F. (2020). Can we cure hepatitis B virus with novel direct-acting antivirals?. Liver Int..

[22] Medas R., Liberal R., Macedo G. (2021). Discontinuation of antiviral therapy in chronic hepatitis B patients. World J. Clin. Cases.

[23] Chang M.L., Liaw Y.F., Hadziyannis S.J. (2015). Systematic review: Cessation of long-term nucleos(t)ide analogue therapy in patients with hepatitis B e antigen-negative chronic hepatitis B. Aliment. Pharmacol. Ther..

[24] Gerlich W.H., Glebe D., Kramvis A., Magnius L.O. (2020). Peculiarities in the designations of hepatitis B virus genes, their products, and their antigenic specificities: A potential source of misunderstandings. Virus Genes.

[25] Tong S., Revill P. (2016). Overview of hepatitis B viral replication and genetic variability. J. Hepatol..

[26] Niklasch M., Zimmermann P., Nassal M. (2021). The Hepatitis B Virus Nucleocapsid-Dynamic Compartment for Infectious Virus Production and New Antiviral Target. Biomedicines.

[27] Papatheodoridi M., Papatheodoridis G. (2019). Can we stop nucleoside analogues before HBsAg loss?. J. Viral Hepat..

[28] Philips C.A., Ahamed R., Abduljaleel J.K., Rajesh S., Augustine P. (2021). Critical Updates on Chronic Hepatitis B Virus Infection in 2021. Cureus.

[29] Feng S., Gao L., Han X., Hu T., Hu Y., Liu H., Thomas A.W., Yan Z., Yang S., Young J.A.T. (2018). Discovery of Small Molecule Therapeutics for Treatment of Chronic HBV Infection. ACS Infect. Dis..

[30] Coffin C.S., Zhou K., Terrault N.A. (2019). New and old biomarkers for diagnosis and management of chronic hepatitis B virus infection. Gastroenterology.

[31] Mak L.Y., Wong D.K., Cheung K.S., Seto W.K., Lai C.L., Yuen M.F. (2018). Review article: Hepatitis B core-related antigen (HBcrAg): An emerging marker for chronic hepatitis B virus infection. Aliment. Pharmacol. Ther..

[32] Martinez M.G., Boyd A., Combe E., Testoni B., Zoulim F. (2021). Covalently closed circular DNA: The ultimate therapeutic target for curing HBV infections. J. Hepatol..

[33] Piermatteo L., Scutari R., Chirichiello R., Alkhatib M., Malagnino V., Bertoli A., Iapadre N., Ciotti M., Sarmati L., Andreoni M. (2022). Droplet digital PCR assay as an innovative and promising highly sensitive assay to unveil residual and cryptic HBV replication in peripheral compartment. Methods.

[34] Gao Y., Li Y., Meng Q., Zhang Z., Zhao P., Shang Q., Li Y., Su M., Li T., Liu X. (2017). Serum hepatitis B virus DNA, RNA, and HBsAg: Which correlated better with intrahepatic covalently closed circular DNA before and after nucleos(t)ide analogue treatment?. J. Clin. Microbiol..

[35] Butler E.K., Gersch J., McNamara A., Luk K.C., Holzmayer V., de Medina M., Schiff E., Kuhns M., Cloherty G.A. (2018). Hepatitis B virus serum DNA and RNA levels in nucleos(t)ide analog-treated or untreated patients during chronic and acute infection. Hepatology.

[36] Wang J., Shen T., Huang X., Kumar G.R., Chen X., Zeng Z., Zhang R., Chen R., Li T., Zhang T. (2016). Serum hepatitis B virus RNA is encapsidated pregenome RNA that may be associated with persistence of viral infection and rebound. J. Hepatol..

[37] Mak L.Y., Yuen M.F. (2018). Letter: Serum HBcrAg is a useful marker for disease monitoring, predicting treatment response and disease outcome of chronic hepatitis B virus infection-authors’ reply. Aliment. Pharmacol. Ther..

[38] Hong X., Luckenbaugh L., Mendenhall M., Walsh R., Cabuang L., Soppe S., Revill P.A., Burdette D., Feierbach B., Delaney W. (2021). Characterization of hepatitis B precore/core-related antigens. J. Virol..

[39] Tseng T.N., Jeng W.J., Hu T.H., Wang J.H., Hung C.H., Lu S.N., Chen C.H. (2023). Combined baseline HBcrAg and end-of-treatment HBsAg predict HBV relapse after entecavir or tenofovir cessation. J. Antimicrob. Chemother..

[40] Carey I., Gersch J., Wang B., Moigboi C., Kuhns M., Cloherty G., Dusheiko G., Agarwal K. (2020). Pregenomic HBV RNA and Hepatitis B Core-Related Antigen Predict Outcomes in Hepatitis B e Antigen-Negative Chronic Hepatitis B Patients Suppressed on Nucleos(T)ide Analogue Therapy. Hepatology.

[41] Adraneda C., Tan Y.C., Yeo E.J., Kew G.S., Khakpoor A., Lim S.G. (2023). A critique and systematic review of the clinical utility of hepatitis B core-related antigen. J. Hepatol..

[42] Pattyn J., Hendrickx G., Vorsters A., Van Damme P. (2021). Hepatitis B Vaccines. J. Infect. Dis..

[43] Fan R., Zhou B., Xu M., Tan D., Niu J., Wang H., Ren H., Chen X., Wang M., Ning Q. (2020). Association between negative results from tests for HBV DNA and RNA and durability of response after discontinuation of nucles(t)ide analogue therapy. Clin. Gastroenterol. Hepatol..

[44] You C.R., Lee S.W., Jang J.W., Yoon S.K. (2014). Update on hepatitis B virus infection. World J. Gastroenterol..

[45] Chan H.L.Y. (2019). Okuda lecture: Challenges of hepatitis B in the era of antiviral therapy. J. Gastroenterol. Hepatol..

[46] Karthikeyan E., Gish R.G. (2025). Strategies to Achieve Functional Cure in HBV: Focus on siRNA. Curr. Hepatol. Rep..

[47] Lu F., Wang J., Chen X., Xu D., Xia N. (2017). Potential use of serum HBV RNA in antiviral therapy for chronic hepatitis B in the era of nucleos(t)ide analogs. Front. Med..

[48] Hall S., Howell J., Visvanathan K., Thompson A. (2020). The Yin and the Yang of Treatment for Chronic Hepatitis B-When to Start, When to Stop Nucleos(t)ide Analogue Therapy. Viruses.

[49] Yan Q., Fu X., Wang Y., Wang G. (2025). Do the therapeutic vaccines hold hope for the treatment of hepatitis B?. Hepatol. Int..

[50] Jansen L., Kootstra N.A., van Dort K.A., Takkenberg R.B., Reesink H.W., Zaaijer H.L. (2016). Hepatitis B virus pregenomic RNA is present in virions in plasma and is associated with a response to pegylated interferon Alfa-2a and nucleos(t)ide analogues. J. Infect. Dis..

[51] Lai C.L., Wong D.K., Wong G.T., Seto W.K., Fung J., Yuen M.F. (2020). Rebound of HBV DNA after cessation of nucleos/tide analogues in chronic hepatitis B patients with undetectable covalently closed. JHEP Rep..

[52] Zhang M., Li J., Xu Z., Fan P., Dong Y., Wang F., Gao Y., Yan J., Cao L., Ji D. (2024). Functional cure is associated with younger age in children undergoing antiviral treatment for active chronic hepatitis B. Hepatol. Int..

[53] Hui R.W., Fung J., Seto W.K., Yuen M.F., Mak L.Y. (2025). Emerging therapies for HBsAg seroclearance: Spotlight on novel combination strategies. Hepatol. Int..

[54] Seto W., Liu K.S., Mak L., Cloherty G., Wong D.K., Gersch J., Lam Y.F., Cheung K.S., Chow N., Ko K.L. (2021). Role of serum HBV RNA and hepatitis B surface antigen levels in identifying Asian patients with chronic hepatitis B suitable for entecavir cessation. Gut.

[55] Wang J., Yu Y., Li G., Shen C., Meng Z., Zheng J., Jia Y., Chen S., Zhang X., Zhu M. (2018). Relationship between serum HBV-RNA levels and intrahepatic viral as well as histologic activity markers in entecavir-treated patients. J. Hepatol..

[56] Rinker F., Zimmer C.L., Höner Zu Siederdissen C., Manns M.P., Kraft A.R.M., Wedemeyer H., Björkström N.K., Cornberg M. (2018). Hepatitis B virus-specific T cell responses after stopping nucleos(t)ide analogue therapy in HBeAg-negative chronic hepatitis B. J. Hepatol..

[57] Hume S.J., Wong D.K., Yuen M.F., Jackson K., Bonanzinga S., Vogrin S., Hall S.A.L., Burns G.S., Desmond P.V., Sundararajan V. (2024). High end-of-treatment hepatitis B core-related antigen levels predict hepatitis flare after stopping nucleot(s)ide analogue therapy. Liver Int..

[58] Fan R., Peng J., Xie Q., Tan D., Xu M., Niu J., Wang H., Ren H., Chen X., Wang M. (2020). Combining Hepatitis B Virus RNA and Hepatitis B Core-Related Antigen: Guidance for Safely Stopping Nucleos(t)ide Analogues in Hepatitis B e Antigen-Positive Patients With Chronic Hepatitis B. J. Infect. Dis..

[59] Komatsu H., Inui A., Fujisawa T. (2017). Pediatric Hepatitis B Treatment. Ann. Transl. Med..

[60] Liu Z., Jin Q., Zhang Y., Gong G., Wu G., Yao L., Wen X., Gao Z., Huang Y., Yang D. (2023). 96-Week Treatment of Tenofovir Amibufenamide and Tenofovir Disoproxil Fumarate in Chronic Hepatitis B Patients. J. Clin. Transl. Hepatol..

[61] Lim S.G., Baumert T.F., Boni C., Gane E., Levrero M., Lok A.S., Maini M.K., Terrault N.A., Zoulim F. (2023). The scientific basis of combination therapy for chronic hepatitis B functional cure. Nat. Rev. Gastroenterol. Hepatol..

[62] Cornberg M., Sandmann L., Jaroszewicz J., Kennedy P., Lampertico P., Lemoine M., Lens S., Testoni B., Wong G.L.-H., Russo F.P. (2025). EASL Clinical Practice Guidelines on the management of hepatitis B virus infection. J. Hepatol..

[63] Liang H., Jiang J., Ren F. (2024). Relationship Between Serum HBV-RNA Levels, Sero-biochemical Indices, and Clinicopathological Parameters in Patients with Hepatitis B Virus. Hepat. Mon..

[64] Song H., Zhang Q., Fang G., Luo X., Wu D., Li H., Zhou K., Zhao X., Xu F., Zhang Y. (2024). Unraveling the Mechanisms of MicroRNA in Suppressing Hepatitis B Virus Progression: A Comprehensive Review for Designing Treatment Strategies. Hepat. Mon..

[65] van Bömmel F., Verbinnen T., Agarwal K., Vanwolleghem T., Lampertico P., Buti M., Janczewska E., Bourliere M., Jezorwski J., Donohue K. (2025). Serum levels of hepatitis B core antibodies and hepatitis B core-related antigen at the time of nucleos(t)ide analog cessation and risk of severe flares in patients with chronic hepatitis B. Hepatol. Commun..

[66] Chi H., Li Z., Hansen B.E., Yu T., Zhang X., Sun J., Hou J., Janssen H.L.A., Peng J. (2019). Serum Level of Antibodies Against Hepatitis B Core Protein Is Associated With Clinical Relapse After Discontinuation of Nucleos(t)ide Analogue Therapy. Clin. Gastroenterol. Hepatol..

[67] Ohlendorf V., Wübbolding M., Gineste P., Höner Zu Siederdissen C., Bremer B., Wedemeyer H., Cornberg M., Maasoumy B. (2022). Low anti-HBc levels are associated with lower risk of virological relapse after nucleos(t)ide analogue cessation in HBe antigen-negative patients. Liver Int..

[68] Wong D.K., Seto W.K., Fung J., Ip P., Huang F.Y., Lai C.L., Yuen M.F. (2013). Reduction of hepatitis B surface antigen and covalently closed circular DNA by nucleos(t)ide analogues of different potency. Clin. Gastroenterol. Hepatol..

[69] Liu Y., Zeng W., Xi J., Liu H., Liao H., Yu G., Chen X., Lu F. (2019). Over-gap PCR amplification to identify presence of replication-competent HBV DNA from integrated HBV DNA: An updated occult HBV infection definition. J. Hepatol..

[70] Caviglia G.P., Abate M.L., Tandoi F., Ciancio A., Amoroso A., Salizzoni M., Saracco G.M., Rizzetto M., Romagnoli R., Smedile A. (2018). Quantitation of HBV cccDNA in anti-HBc-positive liver donors by droplet digital PCR: A new tool to detect occult infection. J. Hepatol..

[71] Raimondo G., Locarnini S., Pollicino T., Levrero M., Zoulim F., Lok A.S. (2019). Update of the statements on biology and clinical impact of occult hepatitis B virus infection. J. Hepatol..

[72] Sosa-Jurado F., Meléndez-Mena D., Rosas-Murrieta N.H., Guzmán-Flores B., Mendoza-Torres M.A., Barcenas-Villalobos R., Márquez-Domínguez L., Cortés-Hernández P., Reyes-Leyva J., Vallejo-Ruiz V. (2018). Effectiveness of PCR primers for the detection of occult hepatitis B virus infection in Mexican patients. PLoS ONE.

[73] Hui R.W., Wong D.K., Mak L.Y., Fung J., Seto W.K., Yuen M.F. (2025). Development and Validation of a High-Sensitivity Droplet Digital PCR Assay for Serum Hepatitis B Virus DNA Detection. J. Viral Hepat..

[74] Musolino C., Cacciola I., Tripodi G., Lombardo D., Raffa G., Alibrandi A., Squadrito G., Raimondo G., Pollicino T. (2018). Behaviour of Occult HBV Infection in HCV-Infected Patients under Treatment with Direct-Acting Antivirals. Antivir. Ther..

[75] Kramvis A., Chang K.M., Dandri M., Farci P., Glebe D., Hu J., Janssen H.L.A., Lau D.T.Y., Penicaud C., Pollicino T. (2022). A roadmap for serum biomarkers for hepatitis B virus: Current status and future outlook. Nat. Rev. Gastroenterol. Hepatol..

[76] Zuccaro V., Asperges E., Colaneri M., Marvulli L.N., Bruno R. (2021). HBV and HDV: New Treatments on the Horizon. J. Clin. Med..

[77] Saitta C., Pollicino T., Raimondo G. (2022). Occult Hepatitis B Virus Infection: An Update. Viruses.

[78] Testoni B., Lebossé F., Scholtes C., Berby F., Miaglia C., Subic M., Loglio A., Facchetti F., Lampertico P., Levrero M. (2019). Serum hepatitis B core-related antigen (HBcrAg) correlates with covalently closed circular DNA transcriptional activity in chronic hepatitis B patients. J. Hepatol..

[79] Chien R.N., Liaw Y.F. (2022). Current Trend in Antiviral Therapy for Chronic Hepatitis B. Viruses.

[80] Navabakhsh B., Mehrabi N., Estakhri A., Mohamadnejad M., Poustchi H. (2011). Hepatitis B Virus Infection during Pregnancy: Transmission and Prevention. Middle East. J. Dig. Dis..

[81] Yi P., Chen R., Huang Y., Zhou R.R., Fan X.G. (2016). Management of mother-to-child transmission of hepatitis B virus: Propositions and challenges. J. Clin. Virol..

[82] Weng D., Zhang C., Wei Q., Zhang L., Zang X., Huang G., Cao Z., Xie Q. (2025). Combination therapies for chronic hepatitis B in the era of emerging novel drugs. Hepatol. Int..

[83] Hou J., Zhang W., Xie Q., Hua R., Tang H., Morano Amado L.E., Yang S.S., Peng C.Y., Su W.W., Chuang W.L. (2024). Xalnesiran with or without an Immunomodulator in Chronic Hepatitis B. N. Engl. J. Med..

[84] Bertoletti A., Le Bert N. (2018). Immunotherapy for Chronic Hepatitis B Virus Infection. Gut Liver.

[85] Dandri M., Petersen J. (2016). Latest developments in the treatment of hepatitis B. Minerva Gastroenterol. E Dietol..

